# Bridging host–guest chemistry with molecule chemistry-covalent organic polyrotaxanes (COPRs): from synthesis to inactivation of bacterial pathogens

**DOI:** 10.1039/d4ra05381h

**Published:** 2024-09-24

**Authors:** Juan Li, Jingsong Yuan, Guoli Sun, Wentao Li, Huihui Hao, Baolong Zhou

**Affiliations:** a Affiliated Hospital of Shandong Second Medical University, Shandong Second Medical University Weifang 261041 Shandong PR China; b Weifang People's Hospital, Shandong Second Medical University Weifang 261000 Shandong PR China; c School of Basic Medicine, Shandong Second Medical University Weifang 261053 Shandong PR China; d School of Pharmacy, Shandong Second Medical University Weifang 261053 Shandong PR China zhoubaolong@sdsmu.edu.cn

## Abstract

As a thriving artificial material, covalent organic frameworks (COFs), boasting inherent structural designability and functional adaptability, and with compositions akin to biological macromolecules, have emerged as a rising star in the field of material science. However, the progression of COFs is significantly impeded by the arduous and intricate preparation procedures of novel building blocks, as well as the inefficient development process of new reactions. An efficient, uncomplicated, and versatile functionalization approach, which has the potential to not only facilitate customized preparation of COFs based on application demands but also enable precise performance control, has become a focal point of research. The formulation of multi-functional COFs through efficient and cost-effective methods poses a critical challenge for the practical application of COFs. This review aims to present the preparation of COFs by amalgamating rigid molecular chemistry with flexible supramolecular host–guest chemistry, adopting a “couple hardness with softness” strategy to meticulously construct intelligent covalent organic polyrotaxanes (COPRs) using conventional reactions. Herein, novel building blocks can be acquired by amalgamating existing macrocycle complexes with framework blocks. The amalgamation of supramolecular chemistry bolsters the capabilities to generate, sense, respond, and amplify distinctive signals, thereby expediting the advancement of multifaceted materials with sophisticated structures. Concurrently, the infusion of supramolecular force endows COPRs with exceptional performance, facilitating multi-mode collaborative antibacterial therapy. This comprehensive review not only promotes the efficient utilization of resources but also stimulates the rapid advancement of framework materials.

## Abstract 2

Covalent organic polyrotaxanes (COPRs), have emerged as thriving artificial intelligent materials, amalgamating the rigid molecular chemistry with flexible supramolecular host–guest chemistry, adopting a “couple hardness with softness” strategy.

## Graphical abstract

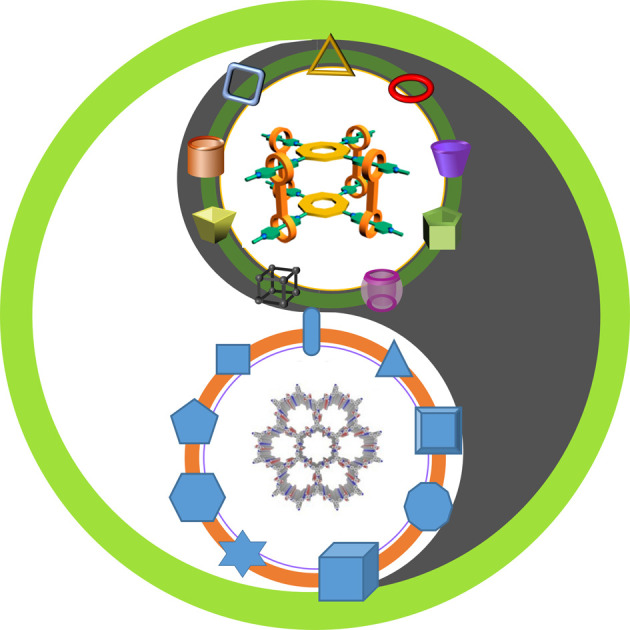

## 1. Introduction

For an extended period, pathogenic bacterial infections, particularly those caused by multi-drug-resistant bacteria due to antibiotic misuse, have posed a significant threat to human health.^1^ The diminishing effectiveness of antibiotics complicates treatment and increases social and economic burdens.^2^ To effectively circumvent clinical issues such as bacterial resistance and variability arising from the excessive use of antibiotics, there is an urgent need to develop safe, effective, and multifunctional antimicrobials that can reduce antibiotic usage.^3^ Despite years of progress in antibacterial agent development, numerous reports highlight various shortcomings of these materials, significantly impeding their clinical application. For instance, metal ion (Ag, Au, Cu, *etc.*) antimicrobials exhibit limited efficacy duration and high biological toxicity. Organic small molecule antibacterial materials are prone to volatilization, posing environmental pollution risks. Semiconductor antibacterial materials face challenges, including complex preparation processes, limited light absorption capabilities, and low efficiency.^4^ Consequently, there is an urgent demand for the targeted development of environmentally friendly, biosafe, and long-lasting smart antibacterial materials.

Recently, covalent organic framework materials (COFs) with both porous and crystalline properties, which are concisely constructed by covalent bonding of organic building blocks, have attracted extensive attention.^5^ As a subclass of porous material with precise structure and function, COFs features the advantages of variety in construction units and synthesis methods, adjustability in pore channels and functions.^6^ The combination of diverse construction units and organic reactions has endowed COFs materials with unlimited research and application possibilities, showing great application prospects in many fields, including the catalysis and biomedicine. With the advent of COFs in 2005, the field has exploded with research and innovation. Over the years, thousands of COFs have been successfully developed, each with unique properties and potential applications.^7^ The development of COFs-based functional materials for biomedical applications has attracted intensive attention.^8^ Compared with other materials, COFs-based antibacterial materials have incomparable advantages. Initially, COFs possesses similar composition to biological macromolecules, ensuring high biosafety for the biomedical application.^9^ Secondly, by adjusting the building blocks or the connection mode between construction units, one can precisely regulate both the electronic structure of the framework and the pore channel microenvironment.^6^ Additionally, the chemical environment surrounding the active site can also be accurately fine-tuned at the molecular or even atomic level. Thirdly, the fully conjugated structure of COFs confers unique photoelectric properties, enabling a reduction in the band gap and consequently enhancing light absorption. As such, COFs offer a versatile platform for integrating structure and performance controllability, greatly facilitating the design and synthesis of COFs tailored for specific applications.

The structure–function relationship stands as the cornerstone of chemistry and materials science.^10^ Currently, the functional diversification of COFs is primarily achieved through two methods: direct construction and post-modification. The direct construction approach involves precise COF building by designing novel blocks or altering connection modes between blocks through reaction screening.^11^ The post-modification enhances functionality by further functionalizing already synthesized COFs *via* controlled reactions.^12^ Consequently, the current focus in COF research lies in topological structure design and functional diversification. However, both methods present challenges. Direct construction faces a lengthy and complex process of monomer design and synthesis.^13^ Meanwhile, post-modification demands careful design of structural blocks with specific reactivity, encountering issues such as quality control difficulties due to low compatibility of functional elements and limited selectivity in organic reaction types. The uncontrollable group introduction rate significantly hinders real-world applications.^14^

In response to these existing challenges, scientists are actively seeking entirely new reactions to synthesize COFs with novel structures and properties. Yet, due to the unique characteristics of COFs, there is a limited selection of reactions that are effectively applicable for their synthesis.^15^ This limitation poses a significant hurdle to the swift progression of COF chemistry.^16^ The primary impetus behind the continued advancement of COFs lies in the development of efficient, straightforward, but widely applicable functional activation methods. These methods offer promise in customizing COFs to align with specific practical needs. In spite of these efforts, precise performance regulation of COFs remains an enduring challenge in scientific research.

Unlike covalent bonds, supramolecular chemistry primarily concerns itself with the assembly system linked by non-covalent and reversible supramolecular interactions, including hydrogen bonding, hydrophilic and hydrophobic associations, electrostatic interactions, and metal coordination.^17^ Dubbed as the “chemistry beyond molecules,” supramolecular chemistry, distinguished by its unique structures and properties, has sparked significant research interest, spanning from architectural innovations to diverse biomedical applications.^18^ Among the various aspects of supramolecular chemistry, supramolecular polymers, particularly those formed through orthogonal self-assembly based on host–guest interactions, have garnered immense attention. These interactions endow these polymers with excellent selectivity and environmental responsiveness. Notably, the supermolecular assemblies preserve the original reactivity of the guest molecules while seamlessly integrating the characteristics of the macrocyclic host.^19^ Consequently, supramolecular self-assembly technology holds immense potential for constructing COF-based functional materials with precise spatio-temporal and stimulus-responsive properties. Although interlocked molecules, such as rotaxanes, formed through host–guest assembly techniques, have been extensively studied, most research focuses on linear polymers.^19*b*^ Developing COFs with novel structures and functions through simple and accessible methods remains a significant challenge.

By incorporating host–guest structures, COFs with distinctive topological structures and properties can be easily synthesized. Nevertheless, current research efforts are primarily focused on chemically modifying macrocycles to achieve specific reactivity, which are then covalently integrated into the porous frameworks.^20^ However, the uniqueness of the macrocyclic structure prevents the direct introduction of many groups. Additionally, the advancement of macrocycle-based COFs faces significant obstacles, including the complexity of the chemical modification process on macrocycle molecules and the low utilization rate of these molecules.^21^ As a result, exploring macrocycle-containing framework materials remains challenging. It is undeniable that expanding structures from points to lines to surfaces can introduce entirely new structures and functions to materials.

Covalent organic polyrotaxanes (COPRs), as a subclass of covalent organic frameworks (COFs), are obtained through the covalent and programmatic assembly of pseudorotaxane units with various building blocks using traditional polymerization reactions. Recently, COPRs have emerged as a new trend in the longitudinal development of COFs, flourishing in the field of materials science.^22^ This novel approach allows for the creation of materials with unique properties and potential applications in various fields such as catalysis, gas capture, and environmental remediation. Different with COFs, covalent organic polyrotaxanes (COPRs) uniquely combine rigid molecular chemistry with flexible supramolecular chemistry through a “couple hardness with softness” strategy, which could realize the precisely construction of intelligent materials. The integration of supramolecular chemistry gives COPRs the ability to generate, sense, respond, and amplify specific signals and stimuli, significantly accelerating the development of multifunctional materials with cutting-edge structures and functions.^23^ Additionally, the introduction of supramolecular forces into COPRs enables special performance characteristics, allowing for multi-mode collaborative antibacterial therapy driven by specific stimuli.^24^ So far, multilevel structured COPRs have been explored as potential candidates for building materials with concurrent novel structures and functions. Recent studies have established a simple and versatile approach for the rational construction of these framework materials. In this review, we aim to provide a comprehensive overview of COPRs, covering commonly used chemical reactions, building blocks, and general synthetic strategies. Furthermore, we summarize the advancements in the preparation and application of COPRs in the antibacterial field, emphasizing the principles and strategies involved in enhancing related activities. Additionally, we prospect the challenges that lie ahead in achieving smart design or formulation of COPRs for advanced applications. Finally, we discuss possible methods and strategies for the simple and efficient preparation of COPRs.

## 2. Types of host–guest complex for the construction of COPRs

After two decades of swift advancement, a diverse array of covalent organic frameworks with distinct building components and linkage patterns have emerged through the amalgamation of numerous organic reactions and synthetic techniques.^25^ Nevertheless, the development of covalent organic polyrotaxanes has been rather limited, primarily due to the narrow selection of host–guest complex structures available. Until now, most host–guest complexes utilized as fundamental units, such as pseudorotaxanes and rotaxanes, have been shaped by electrostatic interactions.^26^ Regrettably, a significant proportion of both natural and synthetic molecules are electrically neutral, posing a significant impediment to the advancement and practical utilization of these materials.^27^

**Fig. 1:**
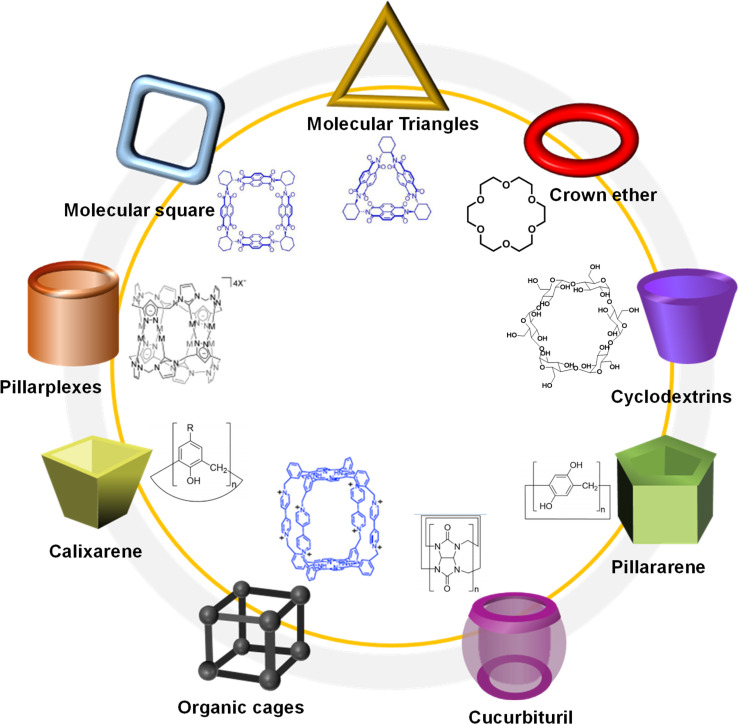
Typical macrocycles with different spatial topological structure for the preparation of host–guest complex.

**Fig. 2:**
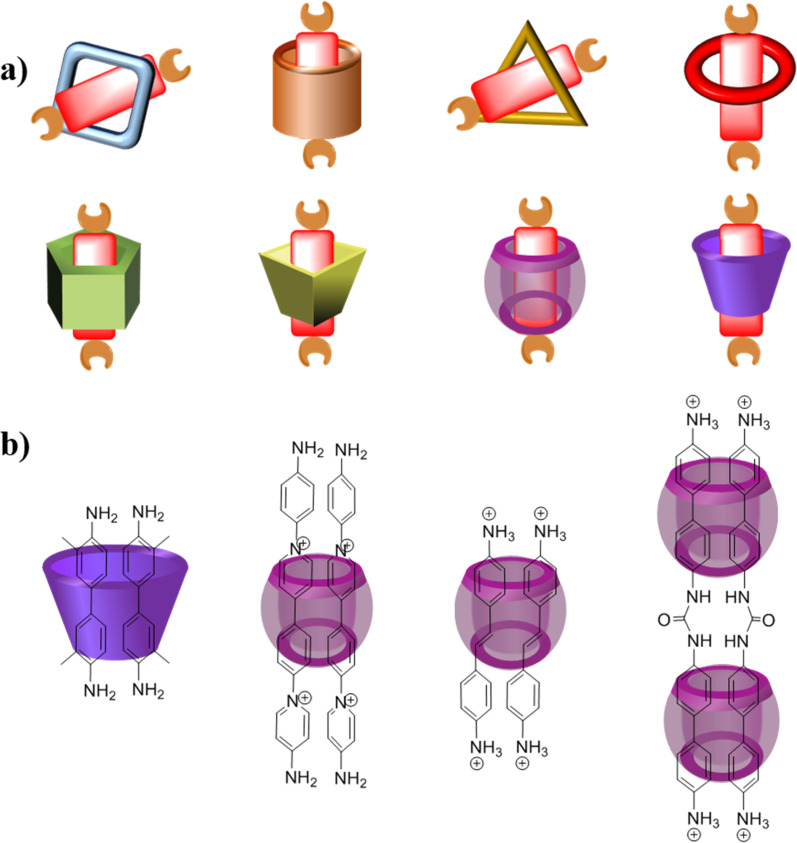
Possible host–guest complex for the construction of COPRs (a) host–guest complex (1 : 1); (b) host–guest complex (1 : 2 and 2 : 2).

Over years of development, a wide range of macrocycles with distinct structures and functionalities have been successfully synthesized, including crown ether, cyclodextrin, cucurbituril, calixarene, organic cages, pillarplexes, pillararene, and more (Fig. 1).^28^ These macrocycles, characterized by their unique cavity structures, can serve as hosts for the formation of inclusion compounds through rational supermolecular self-assembly.^29^ Additionally, there is a plethora of organic building blocks available for the synthesis of COFs, which can function as guest molecules in the formation of host–guest complexes. Thus, the integration of diverse macrocycle libraries with framework block libraries offers limitless opportunities for the creation of novel building blocks through rational supermolecular self-assembly. These novel blocks not only retain their original reactive properties but also introduce new characteristics inherent to the macrocycles (Fig. 2).

## 3. Types of reactions and methods for the construction of COPRs

It is widely recognized that COFs are typically constructed through reversible condensation reactions of molecular building blocks, imparting both crystallinity and thermal stability to these series of material.^30^ Currently, various reactions have proven effective in the fabrication of COFs, such as the Schiff base reactions, boron-based chemistry, Mannich reaction, and Knoevenagel reaction.^5^ Additionally, numerous synthetic strategies have been demonstrated to be viable for COF synthesis, including the ultrasonic, electrochemical, emulsion, solvothermal, as well as the mechanochemical methods.^7^

However, due to the requirement of preserving the stability of the host–guest complex, only a limited number of reactions are appropriate for the synthesis of COPRs.^9^ The stability of the host–guest complex heavily relies on the reaction temperature and the solvent employed. It is commonly acknowledged that the standard approach for synthesizing COFs still heavily depends on solvothermal methods that involve organic solvents, often carried out in sealed tubes at elevated temperatures and pressures. These conditions make the inclusion structure of the complex susceptible to disruption.^6^ To systematically catalog and understand the physicochemical properties of COPR, it is imperative to rigorously screen both the types of reactions and the conditions under which they are performed.

### 3.1 Preparation method selection

According to the structure characteristics of the assembled block, the preparation method should be finely selected. On the one hand, the solvent should be finely controlled to make the host and guest complex in a stable state. On the other hand, the host/guest complex is guaranteed to be in a stable state during polymerization only by low temperature reaction. Therefore, the reaction and preparation methods which could not only effectively avoid the use of not only organic solvent, but also the high reaction temperature, are highly preferred.

In this regard, the mechanochemistry synthesis method, water phase synthesis method, hydrosol-gel synthesis method, or a combination of these methods, particularly the environmentally friendly mechanochemistry synthesis, stand out for the targeted synthesis of COPRs. Different with solvent thermal synthesis, mechanochemistry achieves chemical transformations through the application of mechanical force, such as mechanical grinding or milling. This approach has emerged as a simple and “green” method for preparing COPRs, offering high scalability.^31^ Notably, mechanochemistry synthesis is also recognized as a prominent approach for preparing inclusion compounds, as it ensures the stability of inclusion structures during the polymerization process.

### 3.2 Chemical reaction selection

Polymerization reactions play a pivotal role in the synthesis of COFs, and the selection of these reactions are depended critically on the reaction characteristics of the building blocks. Over the past decades, significant progress has been made in COF synthesis, the availability of reversible chemical reactions remains a key challenge.^32^ Furthermore, the formation of COPRs presents unique challenges due to the simultaneous control of multiple dynamic forces and the confinement effects of macrocycles. These factors make it more difficult to obtain the ideal structure compared to the growth of reversible covalent bonds alone. Thereby, only handful reactions are accessible for the preparation of COPRs. And the scarcity of reversible reactions not only restricts the potential for creating COPRs with tailored properties, but also hinders the exploration of new material designs.

#### 3.2.1 Schiff base reaction

**Fig. 3:**
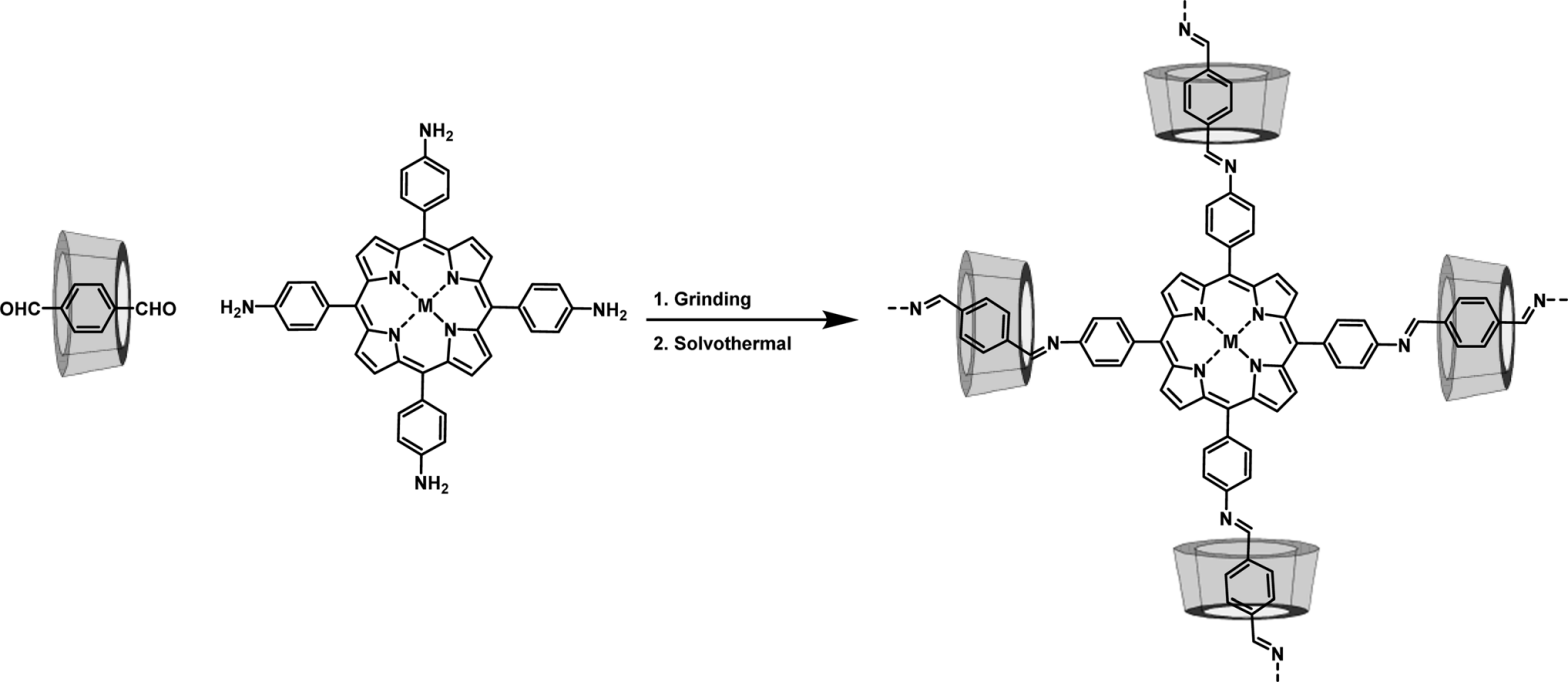
Typical COPRs based on the Schiff-base chemistry.

Dynamic imine based Schiff-base chemistry has been regarded as the state-of-the-art reaction for the synthesis of COFs, with the characteristics of high stability, rich porosity and excellent crystallinity.^33^ Schiff-base chemistry with tunable reaction temperature and various optional reaction solvent, provides perspectives for the targeted preparation of COPRs towards specific applications (Fig. 3). Apart from organic solvents, water or even the solvent free conditions are accessible for the preparation of COFs, which guarantees the stability of complex during the polymerization to get the COPRs with ideal structure and structure.^34^ The inclusion compound containing the multiple aldehyde and amines could be direct used as the building blocks for the targeted preparation of COPRs with specific structure and functions.

#### 3.2.2 Michael addition–elimination reaction

**Fig. 4:**
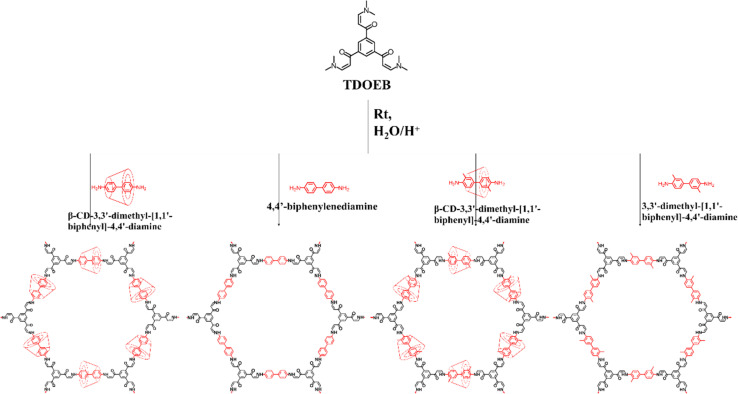
Typical COPRs based on the Michael addition–elimination reaction.

Michael addition–elimination reaction realized *via* the dynamic polymerization of β-ketoenamines and aromatic amines in the s in ambient aqueous systems also displays great application for the synthesis of COPRs.^35^ For the one hand, the inclusion compound structure could be finely retained in the aqueous systems at ambient temperature and pressure. For the other hand, different with the traditional aromatic amines, the aromatic amines inclusion compounds possess a higher solubility in water, which are benefit for the polymerization (Fig. 4). *Via* the structurally diverse β-ketoenols with amines, various COPRs with different structure and function could be facilely prepared.

## 4. Antibacterial application of COPRs

Polyrotaxanes possess numerous advantages that render them exceptionally well-suited for antibacterial applications, surpassing other antibiotics in terms of suitability. Initially, polyrotaxanes exhibit unique structures enabling them to efficiently adhere to bacterial membranes. This direct interaction facilitates the exertion of therapeutic effect precisely to the site of infection, thereby guaranteeing optimal efficacy. Secondly, polyrotaxanes offer greater flexibility in structural design and functional regulation compared to other antibiotics. This versatility allows polyrotaxanes to serve as a multi-functional platform, capable of synergistically amplifying bacterial inactivation. Thirdly, traditional antibiotics are increasingly facing the significant challenge of bacterial resistance. Polyrotaxanes, however, with their distinct mode of action, may provide an alternative route to overcome this resistance. By targeting bacteria through different mechanisms, polyrotaxanes can prove effective against resistant strains that have become immune to conventional antibiotics. Fourthly, antibiotics often cause side effects in humans, ranging from allergic reactions and gastrointestinal distress to more severe health issues. By contrast, polyrotaxanes, owing to their targeted action and precise mechanism, potentially carry a reduced risk of these side effects, thereby offering a safer therapeutic alternative. Finally, polyrotaxanes frequently exhibit antibacterial activity against a broad spectrum of bacteria, encompassing both Gram-positive and Gram-negative strains, rendering them a versatile tool in fighting bacterial infections. In conclusion, polyrotaxanes possess notable advantages in antibacterial applications. Their ability to effectively adhere to bacterial membranes, serve as a multi-functional platform, overcome bacterial resistance, reduce side effects, and exhibit broad-spectrum activity positions them as a promising alternative or adjunct to traditional antibiotics in the treatment of bacterial infections.

### 4.1 Phototherapy antibacterial

Phototherapy-based antibacterial strategies, primarily consisting of photodynamic and photothermal treatments, have sparked significant research interest as a precise, noninvasive, and highly controllable therapeutic approach.^36^ Unlike traditional antibiotics, this emerging phototherapy rarely encounters bacterial resistance, making it a convenient and biocompatible method for efficiently eradicating bacterial infections using light irradiation.^37^

Beyond the role of laser irradiation, photosensitizers (PSs) are crucial in phototherapy.^38^ Porphyrin and its derivatives are the most commonly used PSs for the photo therapy.^39^ However, due to their inherent conjugated structure, the performance of these PSs in phototherapy is significantly hampered by self-quenching caused by severe self-aggregation, particularly in fully conjugated 2D porphyrin-based COF photosensitizers.^40^ To overcome this challenge, innovative preparation methods are urgently needed to fine-tune the layer distance. Furthermore, the high cost of PSs poses a significant obstacle to the widespread application of phototherapy.

**Fig. 5:**
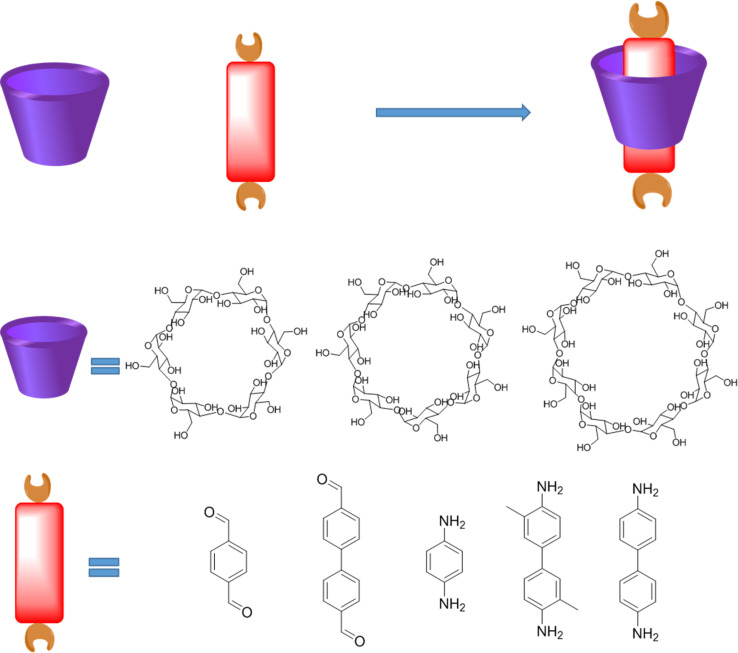
Typical structure of cyclodextrin (CD) host, aromatic guest, as well as corresponding host–guest complex.

Cyclodextrin (CD) macrocycle inclusion compound, with the varied cavity size, has been widely used as building blocks for the preparation of polyrotaxanes (Fig. 5).^41^ In general, the distance of π–π action is about 0.3 nm, and the diameter of the outer cavity of β-CD is 1.4 nm.^42^ Therefore, the structure, packing mode and properties of the material can be effectively controlled *via* the introduction of CD compounds into COPRs. Furthermore, owing to the water solubility of cyclodextrin, CD inclusion compound can remarkably improve the water solubility of original monomer, which could even be used as the monomers for the hydrosol-gel synthesis.^43^ Noteworthily, the macrocycle host also features unique physical and chemistry performance, which empowers special activity to the final COPRs. For example, the crown ether could act as antibacterial agent binding well with the bacterial cell membrane.^44^ Meanwhile, cyclodextrin possesses bactericidal and antiseptic properties, which could be used to improve the biosafety of COFs (Fig. 5).^45^ Meanwhile, numerous reports have demonstrated cucurbituril features excellent antiviral properties.^46^ Thereby, the integration of host–guest complex structure into the COPRs could obtain unexpected improvements in both the structural and performance.

**Fig. 6:**
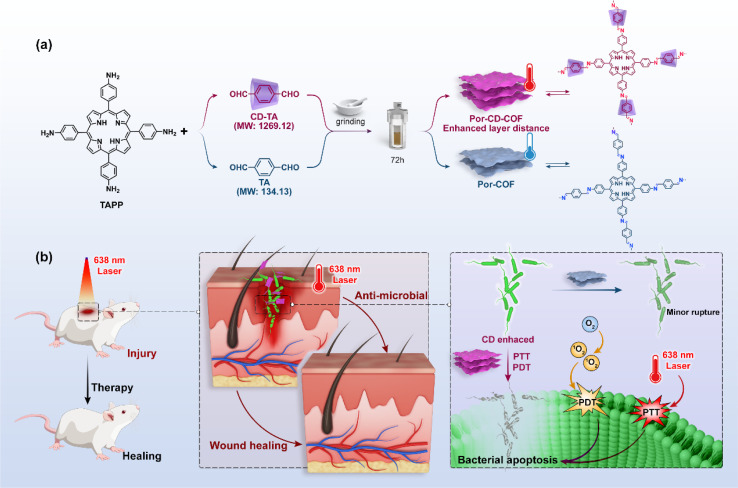
Schematic representation of the synthesis and antimicrobial application of Por-CD-COF. (a) The typical route for the synthesis of Por-CD-COF and the typical structure of Por-CD-COF; (b) schematic antimicrobial mechanism for the Por-CD-COF.

The Zhou group successfully threaded β-CD into the COFs skeleton by sequentially combining mechanical grinding and solvothermal synthesis (Fig. 6). This led to the creation of CD-threaded COFs, termed Por-CD-COF.^9^ This material was effortlessly constructed by integrating dynamic imine-bond formation with host–guest self-assembly. In this process, the pseudorotaxane, formed by the inclusion of aromatic terephthalaldehyde with bactericidal active β-CD, was polymerized with photosensitive tetraminoporphyrin through a combination of mechanical grinding and solvothermal synthesis, utilizing Schiff base chemistry. The incorporation of polyrotaxane units into the COFs skeleton effectively altered the distance between conjugated layers, thereby preventing photo activity quenching caused by the dense aggregation of fully conjugated COF layers. Significantly, the inclusion of low-cost, high molecular weight CD considerably reduced the required amount of photoactive porphyrin monomer. Remarkably, under the same molar amount of reaction monomers, the ideal product weight of Por-CD-COF approaches 5 times that of CD-free COF, significantly reducing the usage of photoactive ingredients.

Meanwhile, evaluations of cytotoxicity and biosafety have shown that the introduction of CDs can significantly enhance the biocompatibility of COPRs.^47^ The synthesized Por-CD-COF exhibited excellent photothermal therapy (PTT) and photodynamic therapy (PDT) activities, surpassing those of CD-free COF.

The threading of CDs in Por-CD-COF not only improves the adhesion of COPRs to bacterial cell membranes, thereby reducing the diffusion distance of reactive oxygen species (ROS) and heat generated under laser irradiation, but also enhances the efficiency of phototherapy. Furthermore, it integrates the intrinsic bacteriostatic properties of CDs with COPRs, resulting in a synergistic amplification of antibacterial capacity. The unique structure of COPRs also effectively mitigates activity quenching caused by severe π aggregation. Consequently, COPRs with CD macrocycles emerge as a highly efficient, material-based antibacterial agent, exhibiting superior photo-synergistic antimicrobial activity against both Gram-positive and Gram-negative bacteria, surpassing counterparts without CD incorporation. Additionally, *in vivo* assays have demonstrated that Por-CD-COF significantly accelerates the healing of bacteria-infected wounds.

**Fig. 7:**
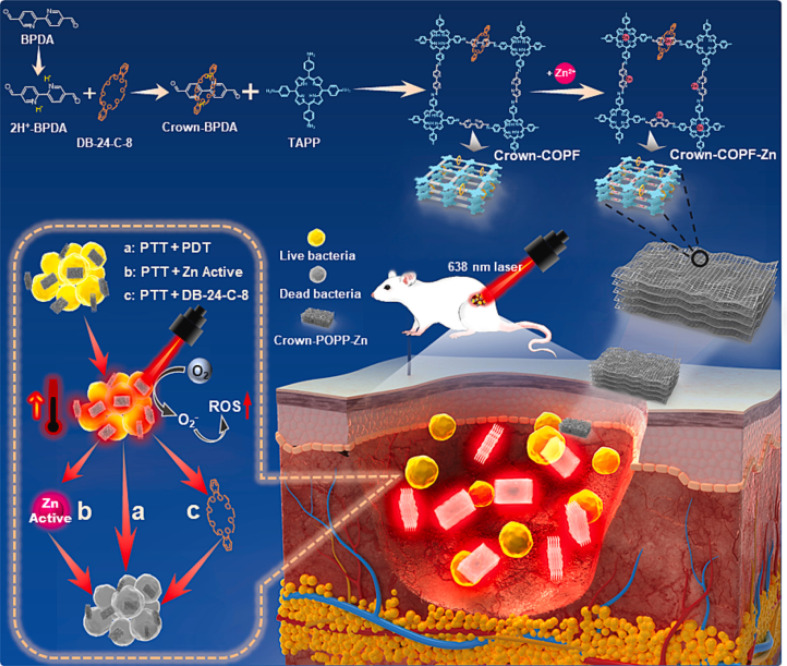
Schematic diagram of the synthesis and antimicrobial mechanism of Crown-COPR-Zn. PTT, PDT and ROS refer to photothermal therapy, photodynamic therapy, and reactive oxygen species, respectively.

Recently, Wang and his colleagues developed a crown ether-based COPRs with mechanical bond, denoted as Crown-COPR, *via* the polymerization between mechanically interlocked molecules (MIMs), specifically, crown-ether threaded rotaxane, with tetramino-porphyrin.^24^ Apart from the alteration of skeleton architecture of COFs, the introduction of mechanical bond also empowers unique property with COPRs. The final Crown-COPR displayed distinguished photosensitive antimicrobial ability towards both the G+ (*Staphylococcus aureus*) and G− (*Escherichia coli*) bacteria, in which the crown ether rings with inherent antibacterial ability could not only improve the adhesion with bacteria, but also effectively adjust the layer distance of COFs layers, remarkably improving the phototherapy sensibility. And the bacterial inactivation activity could be further improved *via* the integration of self-adaptive multinuclear Zn^2+^ center. The COPR with Zn^2+^ impregnation, denoted as Crown-COPR-Zn, featuring self-adaptive multinuclear Zn^2+^ center, could deliver synergistic PDT/PTT/Zn^2+^/Crown ether antimicrobial activity with photo controlled Zn^2+^ release capacity (Fig. 7). *In vivo* assay demonstrated the Crown-COPR-Zn could also effectively promote the repairing of wounds with bacteria-infection.

## 5. Other application of COPRs

Meanwhile, the COPRs could also realize the precise regulation of skeleton polarity, electronic properties, topological structure, stacking mode and biological activity of the material. The COPRs obtained *via* the incorporation of mechanically interlocked molecules (MIMs) into crystalline porous polymer provides access to design materials with not brand new structures and functionalities.^9,24^ For example, the heteroatom rich macrocycles could significantly enhance the skeleton polarity, offering rich active sites for the iodine and carbon dioxide capture. Additionally, supramolecular self-assembly is conducive to obtain the COPRs with high responsiveness towards specific applications.

### 5.1 Carbon dioxide and iodine capture

It is commonly recognized that both the iodine and carbon dioxide capture ability was highly dependent on the heteroatom sites.^48^ The introduction of heteroatomic sites and enhancement of the skeleton polarity have become the general method for improving the properties of adsorbents.

**Fig. 8:**
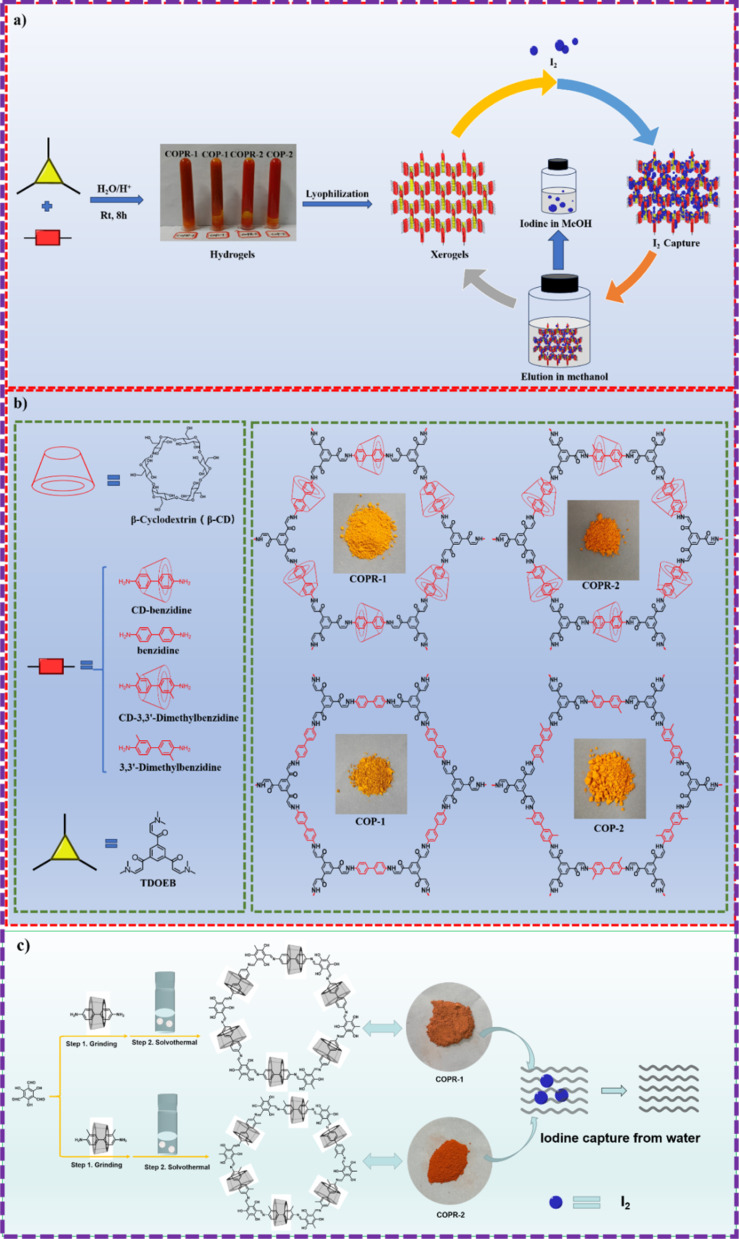
(a) Schematic route for the preparation of COPRs *via* the Michael addition–elimination reaction; (b) representative structure of COPRs obtained from the Michael addition–elimination reaction: (c) schematic route for the preparation of COPRs *via* the Schiff base reaction.

Inspired by this, Zhou and his co-workers employed aqueous phase synthesis at room temperature to successfully produce two cyclodextrin (CD)-threaded covalent organic polyrotaxanes (COPRs), designated as COPR-1 and COPR-2 (Fig. 8a and b).^49^ These innovative COPRs could function as a versatile and recyclable platform that effectively addresses both organic/radioactive iodine pollutants and captures carbon dioxide from the atmosphere. Specifically, COPR-1 and COPR-2 were rapidly synthesized through a Michael addition–elimination reaction between β-ketoenamine and β-CD aromatic amines in water at ambient temperature and pressure, eliminating the need for organic solvents. Notably, the β-CD inclusion significantly boosts the solubility and reactivity of the building blocks in water. The distinctive polyrotaxanes structure endows the COPRs with high CO_2_ affinity, making them suitable as stable solid adsorbents for CO_2_ capture. Additionally, the integration of CD units and aniline-enone linkages within the polymer network allows for the simultaneous grafting of electron donors, such as basic O and N-sites, onto the surface of the COPRs, greatly favoring the capture of Lewis acidic carbon dioxide.

The homogeneous threading of β-CD units throughout the polymer backbone also introduces multiple heteroatom absorption sites, enhancing the polarity of the COPRs skeleton and significantly improving iodine capture capabilities in various environments. This enrichment of absorption sites is further validated through DFT calculations. Consequently, these novel COPRs exhibit unique skeletal structures combining high-polarity CD units, an N-rich backbone, and fully conjugated π-networks, collectively serving as electron-rich binding sites for I_2_ molecules, ultimately achieving exceptional adsorption capacities.

Recently, Zhou and his colleagues devised an innovative yet straightforward approach for the simultaneous design and synthesis of COPRs with customizable structure and functionality (Fig. 8c).^50^ These unique COPRs were created by seamlessly integrating CD-based pseudorotaxane moieties into the COF backbone. This was achieved through a stepwise Schiff base reaction, combining mechanical grinding with solvothermal synthesis. Besides the inherent adsorption sites, the synthesized COPRs also feature abundant O-sites stemming from the evenly distributed threaded CD units across the main chain of polymers. Consequently, these COPRs can serve as highly efficient adsorbents, capable of extracting iodine from water. This groundbreaking work bridges the gap between mechanically interlocked CDs (MIMs) and polymeric materials, paving the way for a cost-effective method to produce COPRs with tailored structures and functions.

The homogeneously threading of the β-CD rings through the carbon skeleton meticulously preserves the original pore structure of the polymer skeletons. This special design scheme not only presents a green, straightforward, and ultra-low-cost method for preparing COPRs with customized structure and function, but also offers direct and effective approaches to achieving carbon neutrality at the source of materials and absorbent production. Undoubtedly, this study can serve as a universal tool for the specific design and modification of COPRs, opening up direct opportunities to fulfill the growing demands of societal development.

### 5.2 Mimicking enzyme catalysis

**Fig. 9:**
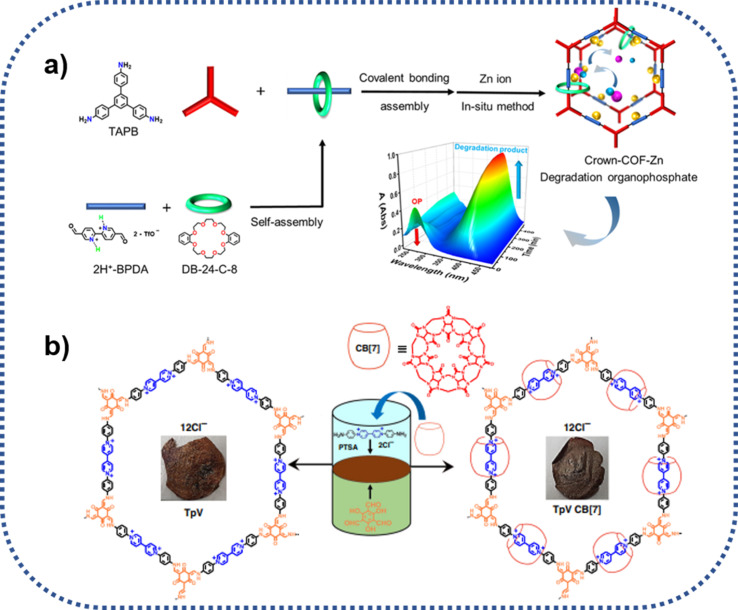
Schematic route for the preparation of crown-ether and cucurbit[7] ring mechanically intercalated COPRs: (a) Crown-COF, and zinc(ii) ion coordinated Crown-COF-Zn; (b) TpVCB[7].

As displayed in Fig. 9a, Zhu group also reported a type of COPR with mechanically interlocked molecules (MIMs) units, denoted as Crown-COF, which could function as molecule machine in response to special external stimulation^51^ In this system, the threaded crown-ether serves as a wheel, while the COF skeleton acts as an axle. Crown-COF, rich in coordination sites including crown-ether coordinated Zn(ii) and bipyridine coordinated Zn(ii), concurrently, could form a metal complex, denoted as Crown-COF-Zn. This special complex demonstrates flexibility, adapting to conformational changes in organophosphorus agents during the hydrolytic process. Notably, Crown-COF-Zn, featuring self-adaptive binuclear Zn^2+^ sites, functions as a highly efficient artificial enzyme, exhibiting exceptional activity for organophosphorus degradation (85.5 μM min^−1^).

### 5.3 Membrane separation

Furthermore, as seen from Fig. 9b, Trabolsi group realized the introduction of mechanical bonds into COPRs using the typical liquid–liquid interfacial condensation method. Different with other methods, the liquid–liquid interfacial reaction induces the formation of 2D polyrotaxaneted film, directly, which hold enormous potential for the specific separation applications.^52^ TpVCB was prepared *via* the copolymerization of diamino-viologen cucurbit[7]uril inclusion compound with 2,4,6-triformylphloroglucinol at the interface of two solvents employing the PTSA catalyzed Schiff base chemistry. Compared with the CB[7] free film, the film of TpVCB[7] presented not only more robust mechanically and thermally stability, but also more luminescent.

## 6. Summary and concluding remarks

COPRs represent a distinctive subclass of artificial multifunctional polymers. They inherently integrate host–guest self-assembly with reversible covalent bond formation, utilizing a clearly structured assembly as the fundamental building blocks. This unique combination showcases significant research value and immense application potential. The extensive range of macrocycle libraries, the accessibility of molecular block libraries, and the diverse preparation techniques offer robust tools for concurrent tuning of porosity, pore channel microenvironments, and functionality. This versatility gives rise to the creation of COPRs with innovative structures and capabilities, fueling the rapid advancement of this field. The “rigid and flexible” approach enables the customization the functions of COPRs, paving the way for a universal and expandable method that introduces multilevel structures into COPRs. This meets the escalating structural complexity and functional demands of framework materials.

Similar to the COFs, COPRs also possess the ‘predesignable’ traits, offering structural adaptability and molecular design flexibility. This provides novel avenues for achieving simplified, cost-effective, and customized synthesis of material-based antibacterial agents. The distinctive attributes of the large ring endow these materials with new functionalities, as evidenced by experimental efforts in designing, synthesizing, and exploring the capabilities of COPRs.

Furthermore, COPRs could also act as a smart platform for crafting multifunctional materials, tailored to specific applications with customized structures and precisely controlled functions. The advancement of COPRs will bolster the diversity of material synthesis methodologies, broaden the molecular structure elements of synthetic chemistry, and enrich the framework synthesis block library to accommodate a wide range of synthesis needs. The availability of various macrocyclic bodies, featuring adjustable cavity sizes and ample modifiable sites, creates opportunities for the introduction of functional groups with customized functions.

This review highlights recent progress in these areas, including molecular structure design, crystallinity enhancement, morphology regulation, and the construction of diverse heterojunctions. Additionally, it underscores recent advancements in their application to water splitting, pollutant degradation, and other photocatalytic reactions. The challenges and future perspectives for exploring cutting-edge photo-catalysts are comprehensively discussed and summarized.

## Data availability

The authors declare that all the experimental date are available.

## Conflicts of interest

There are no conflicts to declare.
